# Transcriptome Analysis of Medicinal Plant *Salvia miltiorrhiza* and Identification of Genes Related to Tanshinone Biosynthesis

**DOI:** 10.1371/journal.pone.0080464

**Published:** 2013-11-19

**Authors:** Lei Yang, Guohui Ding, Haiyan Lin, Haining Cheng, Yu Kong, Yukun Wei, Xin Fang, Renyi Liu, Lingiian Wang, Xiaoya Chen, Changqing Yang

**Affiliations:** 1 Shanghai Chenshan Plant Science Research Center, Shanghai Chenshan Botanical Garden, Chinese Academy of Sciences, Shanghai, China; 2 National Key Laboratory of Plant Molecular Genetics and National Center for Plant Gene Research, Institute of Plant Physiology and Ecology, Shanghai Institutes for Biological Sciences, Chinese Academy of Sciences, Shanghai, China; 3 Key Laboratory of Systems Biology, Shanghai Institutes for Biological Sciences, Chinese Academy of Sciences, Shanghai, China; 4 Shanghai Center for Plant Stress Biology, Shanghai Institutes for Biological Sciences, Chinese Academy of Sciences, Shanghai, China; University of New South Wales, Australia

## Abstract

*Salvia miltiorrhiza* Bunge, a perennial plant of Lamiaceae, accumulates abietane-type diterpenoids of tanshinones in root, which have been used as traditional Chinese medicine to treat neuroasthenic insomnia and cardiovascular diseases. However, to date the biosynthetic pathway of tanshinones is only partially elucidated and the mechanism for their root-specific accumulation remains unknown. To identify enzymes and transcriptional regulators involved in the biosynthesis of tanshinones, we conducted transcriptome profiling of *S. miltiorrhiza* root and leaf tissues using the 454 GS-FLX pyrosequencing platform, which generated 550,546 and 525,292 reads, respectively. RNA sequencing reads were assembled and clustered into 64,139 unigenes (29,883 isotigs and 34,256 singletons). NCBI non-redundant protein databases (NR) and Swiss-Prot database searches anchored 32,096 unigenes (50%) with functional annotations based on sequence similarities. Further assignments with Gene Ontology (GO) terms and KEGG biochemical pathways identified 168 unigenes referring to the terpenoid backbone biosynthesis (including 144 MEP and MVA pathway genes and 24 terpene synthases). Comparative analysis of the transcriptomes identified 2,863 unigenes that were highly expressed in roots, including those encoding enzymes of early steps of tanshinone biosynthetic pathway, such as copalyl diphosphate synthase (SmCPS), kaurene synthase-like (SmKSL) and CYP76AH1. Other differentially expressed unigenes predicted to be related to tanshinone biosynthesis fall into cytochrome P450 monooxygenases, dehydrogenases and reductases, as well as regulatory factors. In addition, 21 *P450* genes were selectively confirmed by real-time PCR. Thus we have generated a large unigene dataset which provides a valuable resource for further investigation of the radix development and biosynthesis of tanshinones.

## Introduction

**Figure 1:**
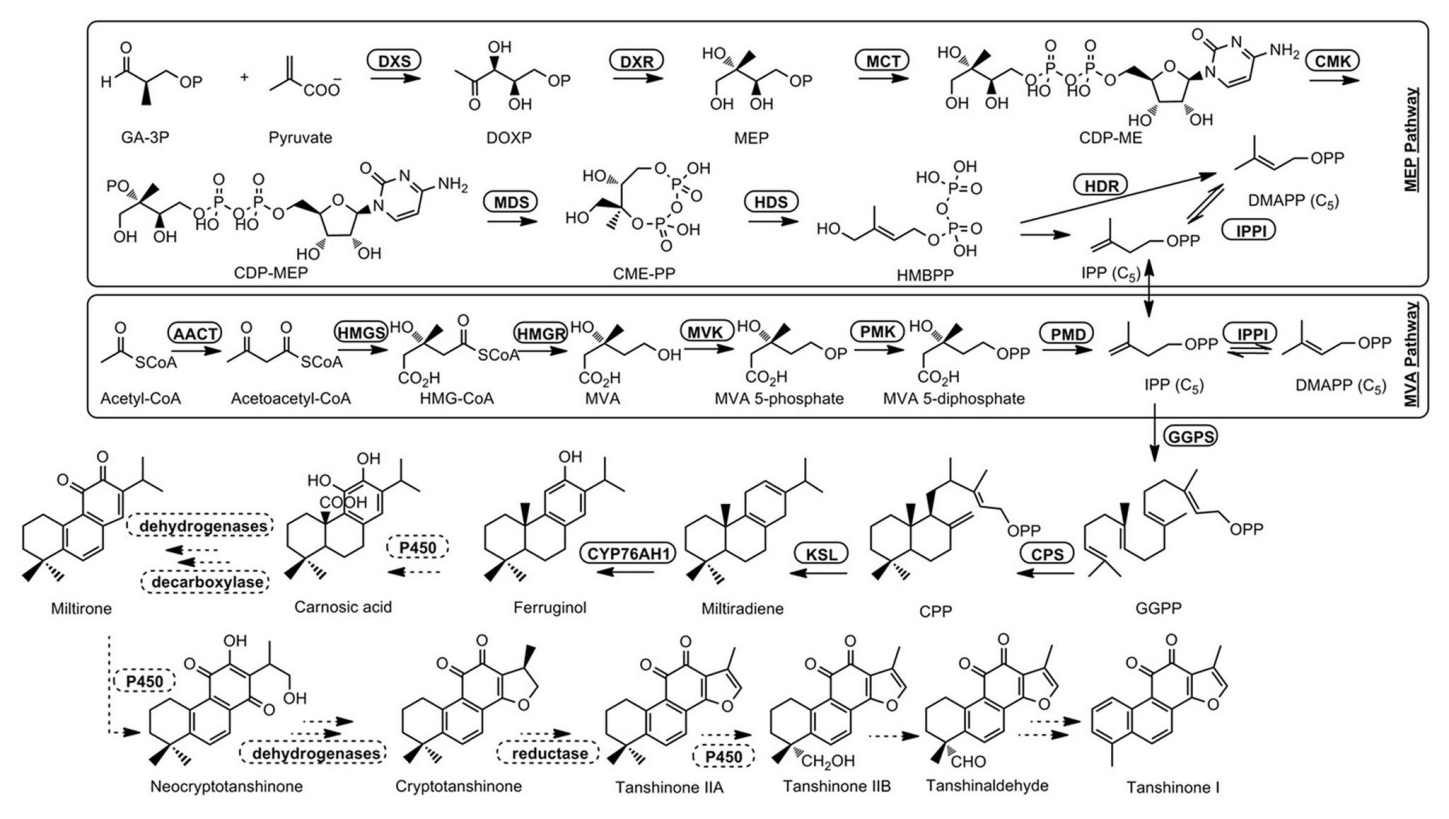
Biosynthetic pathway of tanshinone compounds. The solid arrows denote known steps and the dashed arrows denote hypothetical steps. Modified from Ma et al. and Gao et al. [7,13]. Enzymes of 2-C-methyl-D-erythritol 4- phosphate (MEP) pathway are follows: 1-deoxy-D-xylulose-5-phosphate synthase (DXS), 1-deoxy-D-xylulose-5-phosphate reductoisomerase (DXR), 2-C-methyl-D-erythritol 4-phosphate cytidylyltransferase (MCT), 4-diphosphocytidyl-2-Cmethyl-D-erythritol kinase (CMK), 2-C-methyl-D-erythritol 2,4-cyclodiphosphate synthase (MDS), 4-hydroxy-3-methylbut-2-enyl diphosphate synthase (HDS), 4-hydroxy-3-methylbut-2-enyl diphosphate reductase (HDR); and enzymes of mevalonate (MVA) pathway are acetyl-CoA acetyltransferase (AACT), 3-hydroxy-3-methylglutaryl-CoA synthase (HMGS), 3-hydroxy-3-methylglutaryl-CoA reductase (HMGR), mevalonate kinase (MVK), 5-phosphomevalonate kinase (PMK) and 5-diphosphomevalonate decarboxylase (PMD). Isopentenyl diphosphate isomerase (IPPI) catalyzes the isomerisation of dimethylallyl dihosphate (DMAPP) to isopentenyl diphosphate (IPP) whereas conversion of IPP to geranylgeranyl diphosphate (GGPP) is catalysed by geranylgeranyl diphosphate synthase (GGPPS). Hypothetical tanshinones biosynthetic pathway was deduced by the consideration of identified diterpenoid natural products from *S. miltiorrhiza* [56,57]. These steps involve series hydroxylation, dehydrogenation and reduction reactions catalysed by cytochrome P450s, dehydrogenases and reductases.

*Salvia miltiorrhiza* Bunge is a perennial plant in the genus *Salvia* of the Lamiaceae family. Its dried root or rhizome is called Danshen in traditional Chinese medicine, and was recorded in first pharmaceutical monograph Shennong’s Classic of Materia Medica (A.D. 102-200). *S. miltiorrhiza* has been cultivated throughout Eastern Asia and used to treat and prevent cardiovascular, cerebrovascular, hyperlipidemia and acute ischemic stroke diseases [1]. The active ingredients in *S. miltiorrhiza* are considered to contain both hydrophilic and lipophilic components. The hydrophilic phenolic acids include rosmarinic acid, salvianolic acid B, lithospermic acid and dihydroxyphenyllactic acid or Danshensu, and they may function as antibacterial, anti-oxidative and antiviral reagents [2,3]. And the lipophilic diterpenoid components are generally known as tanshinones, including structurally related tanshinone I, tanshinone IIA, cryptotanshinone, and dihydrotanshinone I. All these diterpenoids share the abietane-type skeletons, and tanshinone IIA is considered to be the most important bioactive component [4,5] (Figure 1).

In plant cells, terpenoids are synthesized through either the cytoplasmic mevalonate (MVA) or the plastidic 2-C-methyl-D-erythritol-4-phosphate (MEP) pathway, with possible cross-talk of the common precursors of isopentenyl diphosphate (IPP) and dimethylallyl diphosphate (DMAPP) [6]. As diterpenoid compounds, the abietane-type tanshinones are proposed to be derived mainly from the MEP pathway, starting from the conversion of geranylgeranyl diphosphate (GGPP) to *ent*-copalyl diphosphate (CPP) and to miltiradiene. After hydroxylation, decarboxylation, oxidation and reduction steps, the miltiradiene is converted into cryptotanshinone, tanshinone I, tanshinone IIA or tanshinone IIB (Figure 1) [7]. 

Based on sequence homology, a number of MVA and MEP pathway enzymes, as well as two diterpenoid pathway enzymes of geranylgeranyl diphosphate (SmGGPPS) synthase and ent-copalyl diphosphate synthase (SmCPS), have been reported [7,8]. However, up to now only two enzymes specific to the tanshinones biosynthetic pathway have been identified: one is the kaurene synthase-like (SmKSL), a diterpene synthase that utilizes copalyl diphosphate (CPP) as substrate to produce miltiradiene [7]; the other is the P450 monooxygenase CYP76AH1, which catalyses the conversion of miltiradiene to ferruginol [9]. It is of great interest to identify enzymes catalysing the remaining steps of the pathway. 

Medicinal plants, which produce different classes of natural products, cover a wide range of plant taxa and most of them have only limited EST, transcriptome and genome data. Next generation sequencing (NGS) platforms provide highly efficient tools to discover novel enzymes and transcription factors from these non-model species. In recent years, genomic approaches have been widely used for discovering and characterizing secondary metabolism pathways and the related genes. For *S. miltiorrhiza*, a cDNA library of whole plantlet was constructed and 10,228 ESTs were generated [10]; a cDNA microarray was used to identify 114 differentially expressed cDNAs in *S. miltiorrhiza* hairy root [11]; 56,774 unigenes in transcriptome of *S. miltiorrhiza* over entire growing cycle were obtained by Illumina deep sequencing [12]; and by searching *S. miltiorrhiza* draft genome (unavailable to public), 40 terpenoid biosynthesis-related genes encoding all enzymes involved in the biosynthesis of universal isoprene precursors of IPP and DMAPP, were proposed [13].

Biosynthesis and accumulation of secondary metabolites are often tissue-specific, and related genes of enzymes and regulators also show organ- or tissue-specific expression patterns [14-16]. Tanshinones are highly enriched in roots, with only a low or trace amount in aerial organs of *S. miltiorrhiza*, such as leaves [17]. Moreover, both the accumulation of diterpenoid tanshinones and expressions of related pathway genes in hairy root cultures of *S. miltiorrhiza* can be induced by biotic (such as the carbohydrate fraction of yeast extract), abiotic (Ag^+^ and Cd^2+^) elicitors, or phytohormones (salicylic acid and methyl jasmonate) [18-23].

Such spatial distribution of tanshinones suggests a root-preferential expression pattern of biosynthetic pathway genes. In contrast to previous approaches that were focused solely on single or mixed tissues, we profiled transcriptomes of root and leaf tissues separately and performed a comparative analysis, which provides a comprehensive insight into tanshinones biosynthesis and regulation, as well as the biology of root (rhizome) development and the secondary metabolism in roots of perennial plants. 

## Results and Discussion

### Transcriptome sequencing, *de novo* assembly and sequence clustering

Tanshinones accumulate mainly in *S. miltiorrhiza* root, with only a trace amount in aerial tissues [17]. To make sure that the root and leaf samples under investigation had a striking difference in tanshinones accumulation, we performed High Performance Liquid Chromatography (HPLC) analyses of their methanol extracts. We found that the levels of tanshinone I, cryptotanshinone, dihydrotanshinone I and tanshinone IIA were high in root, but barely detected in leaf (Figure S1). These results confirmed previous observations and ensured that our root and leaf samples were suitable for subsequent comparative analyses of transcriptomes.

**Figure 2:**
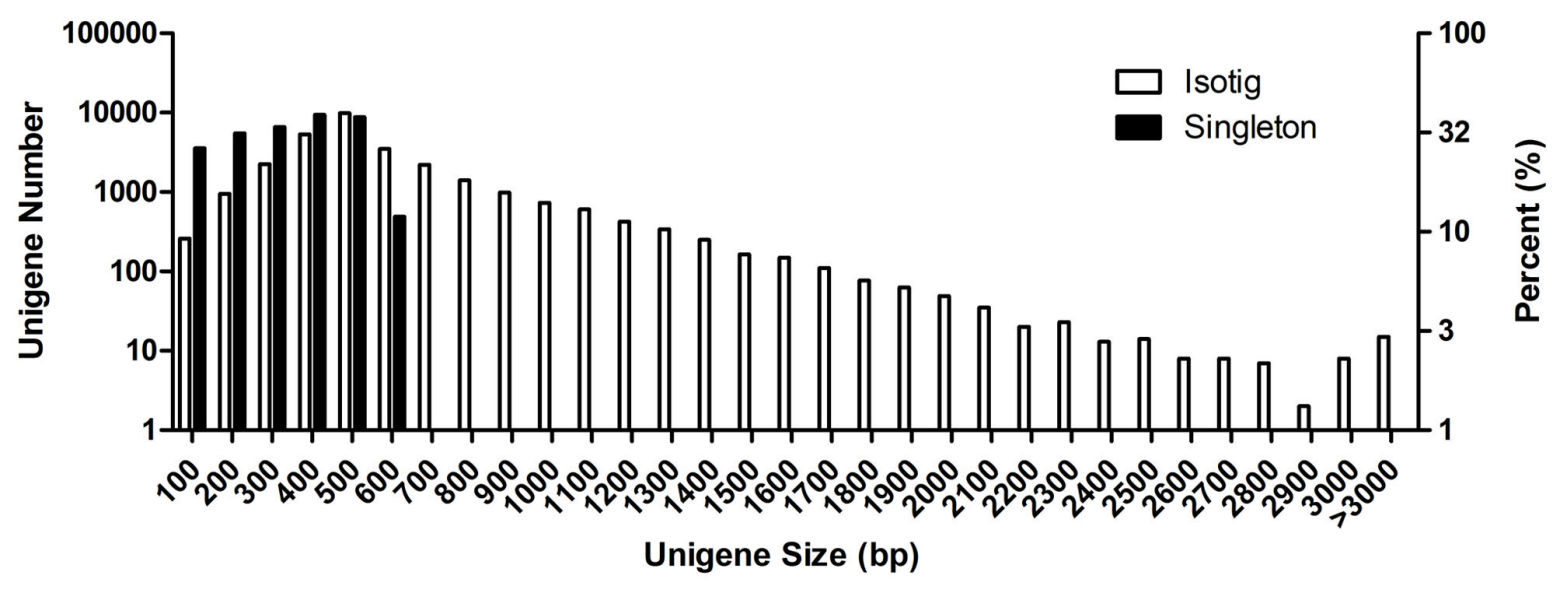
Sequence length distribution of unigenes in the S. *miltiorrhiza* transcriptomes of root and leaf.

The root and leaf RNA samples were profiled by pyrosequencing with Roche’s 454 GS-FLX system, which generated 550,546 and 525,292 reads, with the average lengths of 325 bp and 375 bp, respectively. After trimming the adapter and low quality reads and removing those shorter than 50 bp, 455,552 and 470,863 high quality reads were obtained from root and leaf libraries, respectively. These reads were combined and assembled into 16,806 isotigs (N50 = 750 bp) and 84,116 singletons. Using a sequence similarity cutoff of 95%, the assembled sequences were clustered into 64,139 unigenes (including 29,883 isotigs and 34,256 singletons), with a mean length of 413 bp and total size of 26.4 Mb (Table 1; Figure 2). 

**Table 1:** Summary of sequencing data of *S. miltiorrhiza* root and leaf transcriptomes.

	**Leaf**	**Root**
Total number of raw reads	525,292	550,546
Total number of clean reads	470,863	455,552
Total clean nucleotides (nt)	158,851,743	141,393,767
Total number of isotigs	16,806	
N50 of the isotigs	750 bp	
Total number of singletons	84,116	
Total number of unigenes	64,139	
Average unigene length	413 bp	

### Functional annotation and pathway analysis

Unigenes were searched against the NCBI non-redundant protein database (NR) and the Swiss-Prot database using blastx program with E-value cut-off of 1e^-5^. Totally 32,010 unigenes had at least one match to known protein sequences in the NCBI NR database and 18,201 unigenes found hits in the Swiss-Prot database. After consolidation, 32,096 unigenes (50%) were functional annotated.

**Figure 3:**
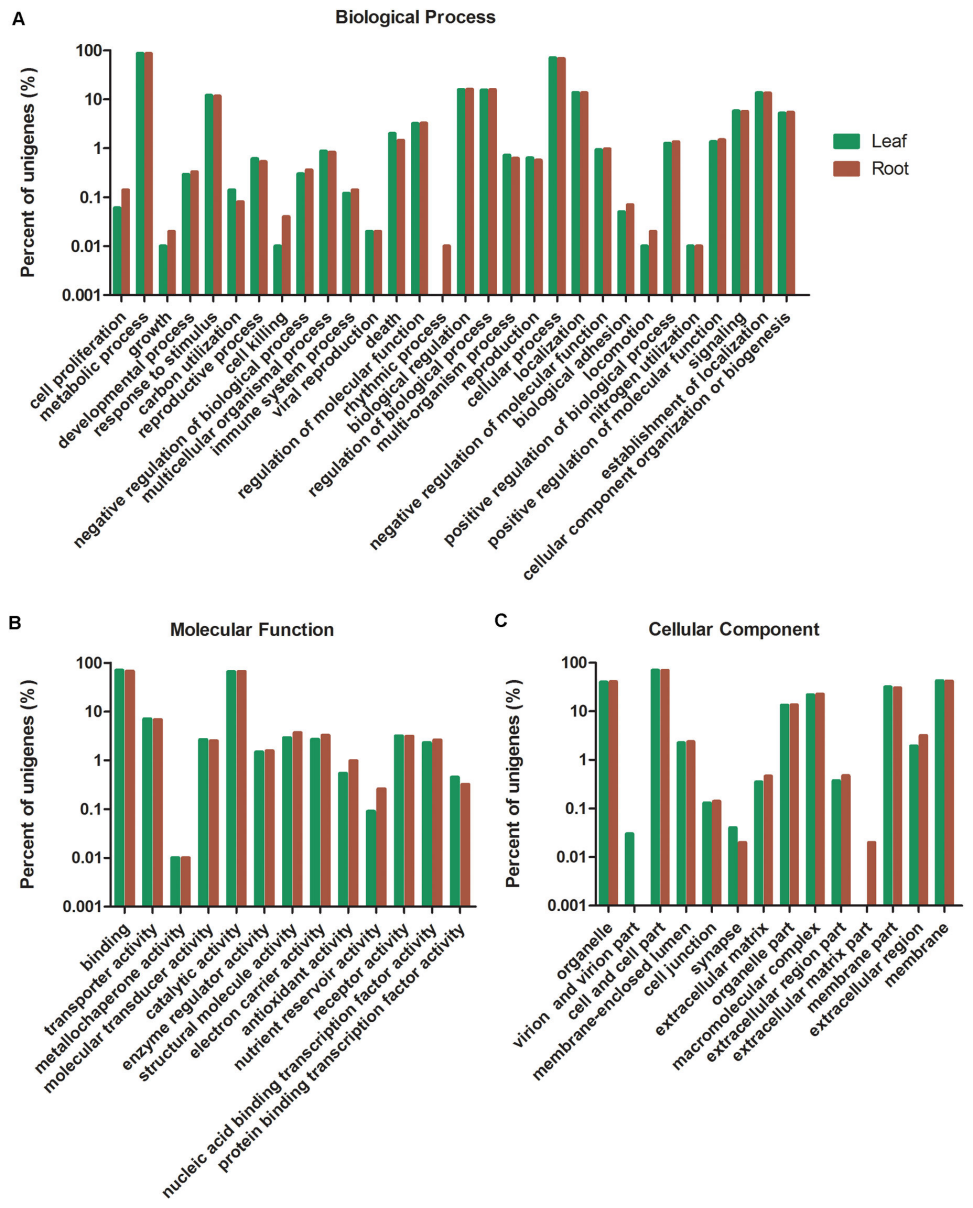
Functional classification of unigenes expressed in *S. miltiorrhiza* leaf and root. Gene Ontology (GO) terms are summarized in three main categories of biological process (A), molecular function (B) and cellular component (C).

Gene Ontology (GO) assignment was performed to functional categorize these annotated unigenes, which resulted in 24,394 unigenes mapped to at least one GO term, of which 21,364 were assigned to the “biological process”, 23,136 to the “molecular function”, and 10,662 to the “cellular component”. Based on origins of the assembled reads, from root or leaf pools, the enrichment of unigenes to GO terms was analysed. The counts for the most categories were similar in root and leaf libraries, such as in the category of “biological process”, ‘metabolic process’ was dominant (86.56% and 85.99% in leaf and root, respectively, the same below), and ‘cellular process’ also had a high-percentage (70.53% and 67.61%); regarding to the “molecular function” category, ‘binding’ (70.29% and 67.48%) and ‘catalytic activity’ (65.48% and 65.88%) were the most enriched; within the category of “cellular component”, ‘cell and cell part’ (69.66% and 68.81%) was the most highly enriched (Figure 3). On the other hand, unigenes classified to the terms of ‘nutrient reservoir activity’, ‘rhythmic process’, ‘locomotion’ and ‘extracellular matrix part’ were more abundant in root (percentage > 2 fold), whereas those related to ‘virion and virion part’ were more in leaf (Figure 3).

Kyoto Encyclopedia of Genes and Genomes (KEGG) pathway assignments were then performed to provide alternative functional annotations to enzymes of biochemical pathways and their Enzyme Commission (EC) numbers. This analysis resulted in 802 unigenes referring to the biosynthesis of secondary metabolites, of which 168 to terpenoids and 389 to other metabolites, including flavonoids and alkaloids (Table S1). 

### Differential expression analysis

We calculated the normalized expression values (RPKM, Reads Per Kilobase per Million mapped reads) of each unigene in root and leaf libraries, and those with > 2-fold change with a false discovery rate (FDR) < 0.01 were considered as differentially expressed genes. Totally 2,863 unigenes were found to have more abundant transcripts in root and 2,561 in leaf, of which 831 and 852 were annotated, respectively. 

Among the differentially expressed genes, those function in chloroplast development and photosynthesis exhibited the most significant differential expression levels between the root and leaf tissues (FDR < 0.01). Genes that encode enzymes of the chlorophyll biosynthesis pathway (such as chlorophyll synthase), chlorophyll A/B binding proteins, and components of photosystem I/II and light-harvesting complex were highly expressed in leaf, but not expressed in root. Similarly, the genes involved in carbohydrate metabolism, such as glucose-1-phosphate adenylyltransferase and glucose-6-phosphate 1-dehydrogenase, and fatty acid metabolism, such as beta-ketoacyl-ACP synthase II, acyl-[acyl-carrier-protein] desaturase, acyl-CoA thioesterase), were also expressed at much higher levels in leaf than in root, in agreement with the fact that plant leaf is the centre for biomass and energy production (Table S2). 

On the contrary, transcripts for transport proteins, including those specific or non-specific for sugars, irons and lipids, were highly abundant in root, which is consistent with their functions in nutrient absorption. Moreover, transcripts of putative pathogen resistance genes were also detected in root, suggesting an active interaction between root and soil organisms (Table S3).

### Isoprenoid biosynthesis

Full length cDNAs of isoprenoid biosynthetic enzymes, including those of MVA and MEP pathways, as well as isoprenyl diphosphate synthases of geranyl diphosphate synthase (GPPS), farnesyl diphosphate synthase (FPPS) and geranylgeranyl diphosphate synthase (GGPPS) [13], were searched against both leaf and root transcriptome libraries to estimate their expression levels. Transcripts of all these genes were detected in both libraries (Table 2). In general, transcripts of MEP pathway genes were more abundant in leaf, as revealed by much higher numbers of reads of *1-deoxy-D-xylulose 5-phosphate synthase* 1 (*SmDXS*1), *1-deoxy-D-xylulose 5-phosphate reductoisomerase* (*SmDXR*) and *4-hydroxy-3-methylbut-2-enyl diphosphate reductase* 2 (*SmHDR*2) genes in leaf than in root. In contrast, the genes in the MVA pathway were more actively expressed in root according to numbers of reads mapped to genes for *acetyl-CoA C-*acetyltransferase 1 (*SmAACT*1), *3-hydroxy-3-methylglutaryl-CoA reductase* (*SmHMGRs*) and *isopentenyl diphosphate isomerase* 1 (*SmIPPI*1) (Table 2). Moreover, some of the genes belonging to the same family showed distinctive expression patterns. For example, of the four *SmDXS* genes analysed, *SmDXS1* was specifically expressed in leaf, whereas *SmDXS2* was up-regulated in root (Table 2), suggesting their functional specialization during evolution.

**Table 2:** Expression of unigenes in isoprenoid biosynthesis pathways.

**Pathway**	**Gene name**	**Unigene**	**Reads in leaf**	**Reads in root**
MVA	AACT 1	isotig00545	4	54
	AACT 2	isotig22400	0	2
		isotig27922	3	0
	HMGS	isotig01307	23	49
	HMGR 1	isotig04635	7	18
	HMGR 2	isotig01508	2	43
	HMGR 3	isotig03994	22	147
		isotig07175	14	126
		gen_HITXW8201CK83K	0	1
		gen_HITXW8201BFCSU	0	1
	HMGR 4	isotig07145	31	484
	MVK	isotig10296	11	4
	PMK	isotig21613	0	2
		gen_HITXW8201A60UW	0	1
	PMD	isotig02363	0	27
	IPPI 1	isotig01015	21	146
	IPPI 2	isotig34210	2	0
MEP	DXS 1	isotig00083	1171	1
	DXS 2	isotig11491	0	26
		isotig10422	0	11
	DXS 3	isotig28922	2	1
		isotig11378	4	14
	DXS 4	isotig00662	7	5
		isotig29585	4	0
	DXS 5	—		
	DXR	isotig06138	31	3
		isotig09197	13	1
	MCT	isotig01252	11	16
		ye_HN6LTMS01DEI5U	1	0
	CMK	isotig00089	36	50
	MDS	isotig11395	7	4
	HDS	isotig00067	98	128
	HDR 1	isotig03934	5	13
		gen_HITXW8201BTHGM	0	1
		gen_HITXW8201EN94F	0	1
	HDR 2	isotig04251	89	0
		isotig11365	17	0
		isotig01210	106	0
Monoterpene	GPPS LSU	isotig05039	2	69
		isotig20070	0	3
		gen_HITXW8201D6C8K	0	1
	GPPS SSUII 1	gen_HITXW8201DT9BE	0	1
	GPPS SSUII 2	isotig10575	44	44
		isotig10082	232	72
		isotig27077	6	0
Sesquiterpene	FPPS	isotig00761	26	65
Diterpene	GGPPS 1	isotig04334	95	324
	GGPPS 2	isotig22076	0	2
	GGPPS 3	isotig10232	19	468

MVA, mevalonate pathway; AACT, acetyl-CoA acetyltransferase; HMGS, 3-hydroxy-3-methylglutaryl-CoA synthase; HMGR, 3-hydroxy-3-methylglutaryl-CoA reductase; MVK, mevalonate kinase; PMK, 5-phosphomevalonate kinase; PMD, 5-diphosphomevalonate decarboxylase; IPPI, Isopentenyl diphosphate isomerase; MEP, Enzymes of 2-C-methyl-D-erythritol 4-phosphate pathway; DXS, 1-deoxy-D-xylulose-5-phosphate synthase; DXR, 1-deoxy-D-xylulose-5-phosphate reductoisomerase; MCT, 2-C-methyl-D-erythritol 4-phosphate cytidylyltransferase; CMK, 4-diphosphocytidyl-2-Cmethyl-D-erythritol kinase; MDS, 2-C-methyl-D-erythritol 2,4-cyclodiphosphate synthase; HDS, 4-hydroxy-3-methylbut-2-enyl diphosphate synthase; HDR, 4-hydroxy-3-methylbut-2-enyl diphosphate reductase; GPPS, geranyl diphosphate synthase; FPPS, farnesyl diphosphate synthase; GGPPS, geranylgeranyl diphosphate synthase.

In plant cell, monoterpenes are synthesized in plastid using geranyl diphosphate (GPP) as precursor, whereas sesquiterpenes and triterpenes are produced in cytoplasm using farnesyl diphosphate (FPP) as precursor. The *GPPS* (both large and small subunits) and *FPPS* genes exhibited similar expression levels in leaf and root. However, transcripts of *geranylgeranyl diphosphate synthase* (*GGPPS*), which produces the precursor for diterpenoids, including tanshinones, were highly abundant and enriched in root (Table 2), with the RPKM values of *SmGGPPS1* being 910.88 in root and 258.41 in leaf; 9.51 in root and 0 in leaf for *SmGGPPS2*; and 1988.85 in root and 78.33 in leaf for *SmGGPPS3*, respectively.

### Terpene synthases

Plants produce a rich array of terpenoids with diverse structures, which play important roles in both basic biological processes and interactions with environmental factors. Terpenoids are synthesized by terpene synthases (TPSs), which comprises of mono-, sesqui- or di-terpene synthases. According to phylogenetic relationships, TPSs are also classified into TPS-a, b, c, d, e/f, g and h subfamilies [24].

From the dataset 24 TPS unigenes were identified. Based on sequence homology to functionally characterized TPSs in the NCBI NR database, 13 unigenes were annotated as (3S)-linalool synthase, myrcene/ocimene synthase or 1.8-cineole synthase, which are all monoterpene biosynthesis [25-30]; and 7 as α-humulene/β-caryophyllene synthase or (+)-α-barbatene synthase, which are sesquiterpene biosynthesis [31-34]. In addition, one copy of ent-kaurene synthase (a diterpene synthase) and 3 fragments of squalene synthases (triterpene synthase) were also identified. 

Most of these TPS genes had low levels of transcripts in both root and leaf libraries. Comparatively, 4 monoterpene synthases (isotig 01344, 05322, 05141, 12563) and 1 sesquiterpene synthase (isotig 08178) showed higher expression levels in root, whereas 1 monoterpene synthase (isotig 07874) and 2 sesquiterpene synthases (isotig 34119, 14598) in leaf (Table 3). 

**Table 3:** Terpene synthase genes of *S. miltiorrhiza*.

**Terpene synthase**	**Unigene ID**	**Length(bp)**	**No. in Leaf**		**No. in Root**	**Annotation**	**Gene bank accession ID**
Mono-	isotig21756	465	0	2		(3S)-linalool synthase	CAD10147
	ye_HN6LTMS01EHVGF	462	1	0		(3S)-linalool synthase	CAD57081
	isotig05322	711	0	9		myrcene/ocimene synthase	NP_189209
	isotig05141	725	1	176		myrcene/ocimene synthase	ABD77416
	isotig12563	450	2	13		myrcene/ocimene synthase	EEE79235
	ye_HN6LTMS01EP8XE	391	1	0		myrcene/ocimene synthase	AES84848
	ye_HN6LTMS01BJR9J	442	1	0		myrcene/ocimene synthase	ACM89961
	ye_HN6LTMS01AIOKP	459	1	0		myrcene/ocimene synthase	ABU87404
	isotig07874	596	15	0		myrcene/ocimene synthase	AFI47927
	isotig01344	1206	0	38		1.8-cineole synthase	EEF01161
	isotig20942	396	0	2		1.8-cineole synthase	ABD77416
	ye_HN6LTMS01DSMZC	425	1	0		1.8-cineole synthase	ACN42009
	ye_HN6LTMS01BY84P	453	1	0		1.8-cineole synthase	ABP01684
Sesqui-	isotig08178	576	0	13		α-humulene/β-caryophyllene synthase	AAX16076
	ye_HN6LTMS01E1WK7	389	1	0		α-humulene/β-caryophyllene synthase	CBI18625
	ye_HN6LTMS01CQ9FP	451	1	0		α-humulene/β-caryophyllene synthase	ADK73618
	isotig30406	378	2	0		α-humulene/β-caryophyllene synthase	ADK73618
	ye_HN6LTMS01BB18N	440	1	0		α-humulene/β-caryophyllene synthase	ADV24747
	isotig14598	342	7	0		α-humulene/β-caryophyllene synthase	ADK73619
	isotig34119	454	7	0		(+)-α-barbatene synthase	CAH10288
Di-	isotig23951	419	1	1		ent-kaurene synthase	CBI32839
Tri-	gen_HITXW8201A8A9N	425	0	1		squalene synthases	AFK29284
	gen_HITXW8201DAZL3	361	0	1		squalene synthases	AAV58897
	gen_HITXW8201A3HRU	406	0	1		squalene synthases	AER23670

### Prediction of candidate enzymes in tanshinone biosynthetic pathway

Because tanshinones are highly accumulated in root, the genes encoding enzymes of tanshinones biosynthesis are expected to show preferential, if not exclusive, expression in root. We first examined the expression pattern of known enzymes in the pathway. Early steps of tanshinones biosynthesis has been partially characterized: the precursor GGPP is converted to CPP by SmCPS [7], then to miltiradiene by the diterpene synthase kaurene synthase-like (SmKSL) enzyme [7], followed by hydroxylation to ferruginol by a P450 monooxygenase, CYP76AH1 [9]. As expected, the transcripts of *SmCPS*, *SmKSL* and *CYP76AH1* were apparently more abundant in root than in leaf. While *SmCPS* and *SmKSL* genes were specifically expressed in root, the *CYP76AH1* expression in root was 3.7-fold higher in root than in leaf (Table 4).

**Table 4:** Known and candidate genes involved in tanshinones biosynthesis.

**Gene**	**Unigene ID**	**RPKM in leaf**	**RPKM in root**	**Product**
*Cpalyl diphosphate synthase* (SmCPS)	isotig01513	0	241.03	Cpalyl diphosphate (CPP)
*Kurene synthase-like* (*SmKSL*)	isotig26056	0	16.34	Miltiradiene
*SmCYP76AH1*	isotig05166	5.88	27.37	Ferruginol
*Cytochrome P450s*	isotig06040	0.00	173.28	Unknown
	isotig11627	4.45	275.21	
	isotig06191	3.20	701.62	
	isotig06719	0.00	688.94	
	isotig00280	59.77	352.42	
	isotig04519	69.75	463.00	
	isotig04821	89.05	491.53	
	isotig13353	15.26	330.35	
	isotig00944	11.25	170.66	
	isotig12561	47.05	451.06	
	isotig04473	41.63	334.71	
	isotig00674	21.93	135.62	
	isotig12886	4.87	245.79	
	isotig01966	0.00	88.54	
	isotig14439	5.95	276.17	
	isotig04633	0.00	113.34	
	isotig04603	0.00	109.85	
	isotig03664	22.73	161.40	
	isotig05120	26.50	151.73	
	isotig13720	10.53	173.60	
	isotig15194	14.96	238.98	
	isotig01982	0.00	47.49	
	isotig10408	0.00	90.47	
	isotig09263	27.23	156.36	
	isotig09616	0.00	81.78	
	isotig05760	12.52	93.56	
	isotig03639	5.04	59.80	
	isotig05574	15.22	94.16	
	isotig13458	0.00	85.12	
	isotig12292	4.64	86.16	
	isotig09252	7.76	80.04	
	isotig14674	6.29	103.70	
	isotig01290	17.46	61.18	
	isotig12891	63.55	196.53	
	isotig09542	0.00	49.01	
	isotig13691	0.00	59.97	
	isotig12983	0.00	55.56	
	isotig12207	0.00	52.54	
	isotig13148	0.00	56.47	
	isotig10175	4.11	59.27	
	isotig01300	10.51	43.33	
	isotig03088	9.42	48.55	
	isotig09140	0.00	39.80	
	isotig06948	0.00	34.65	
	isotig25188	0.00	48.18	
	isotig13449	0.00	53.07	
	isotig12432	0.00	48.39	
	isotig11783	4.50	55.62	
	isotig11894	9.07	65.45	
	isotig09674	19.96	82.29	
	isotig02125	38.90	86.53	
	isotig06295	6.45	43.21	
	isotig13770	0.00	44.05	
	isotig11385	22.16	82.22	
	isotig12590	4.75	48.93	
	isotig05973	12.63	9559.75	
	isotig00853	37.86	2210.09	
	isotig09109	354.60	969.42	
	isotig12661	0.00	34.87	
	isotig08939	0.00	561.04	
	isotig00890	9.52	21.27	
	isotig07498	6.98	57.53	
*Double band reductase* (SmDBR)	Isotig01665	136.05	313.59	Unknown
*Short-chain dehydrogenase* (SmSD)	Isotig06117	6.34	98.08	Unknown

**Figure 4:**
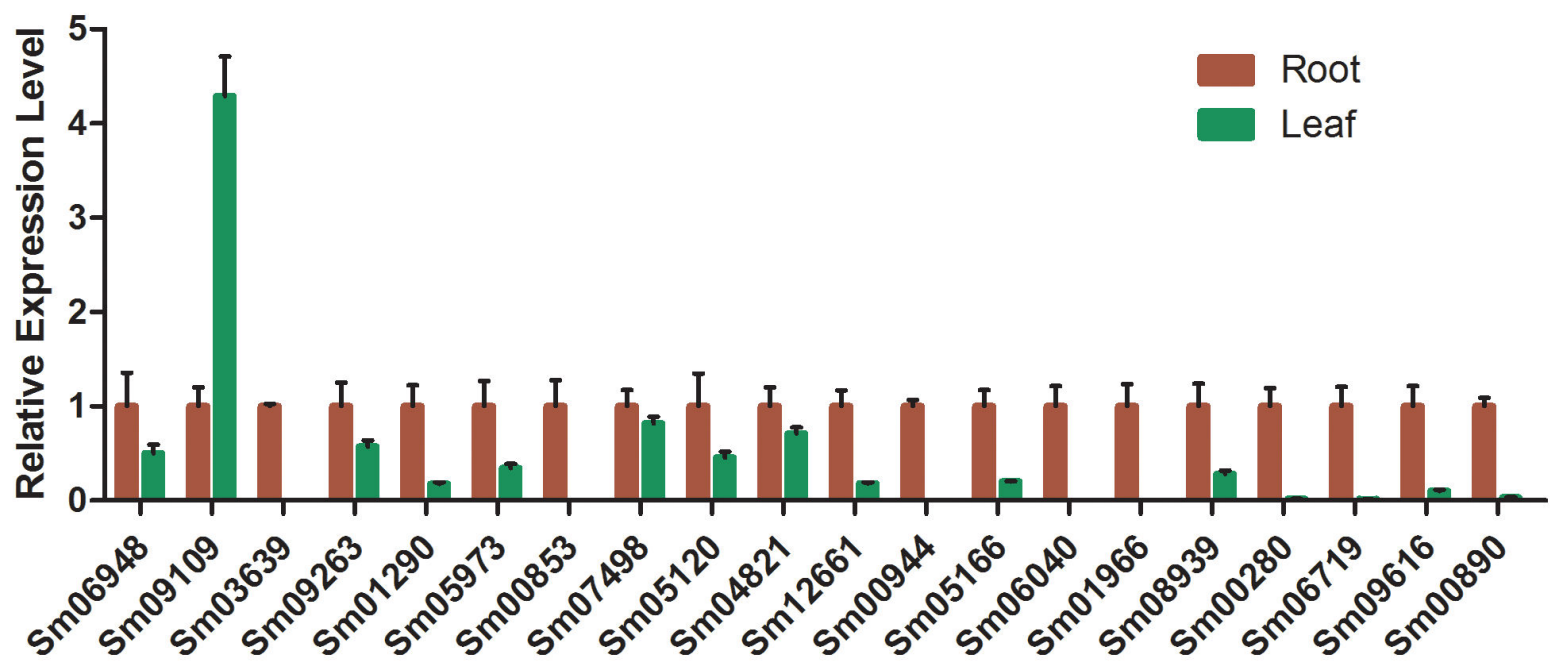
Relative expression levels of P450 genes in *S. miltiorrhiza* root and leaf. Total RNAs were extracted from root and leaf of 1-year-old *S. miltiorrhiza* and expression levels of *P450s* were analysed by quantitative real-time PCR. *ACTIN* was used as the internal reference gene and the relative abundance of each P450 genes in roots were compared to those in leaf, which were all set to be 1. Error bars indicate standard deviations of three biological replicates.

Cytochrome P450s are widely involved in the biosynthesis of secondary metabolites , including phenolic compounds, flavonoids, isoprenoids and alkaloids [35]. In our hypothetical tanshinones biosynthetic pathway, several steps are deduced to be catalysed by P450s (Figure 1). To identify additional candidate genes, RPKM values of *P450* family unigenes in the two libraries were compared, which resulted in 63 *P450* unigenes with > 2-fold (FDR < 0.05) expression levels in root than in leaf (Table 4). To validate this, 21 *P450* unigenes were selected and their expression patterns were analysed by quantitative real-time RT-PCR (qRT-PCR), of which 20 showed higher expression levels in root, and only one showed a reverse pattern (Figure 4). These data suggested that the expression patterns deduced from the RPKM values in our transcriptome analyses are reliable. 

**Figure 5:**
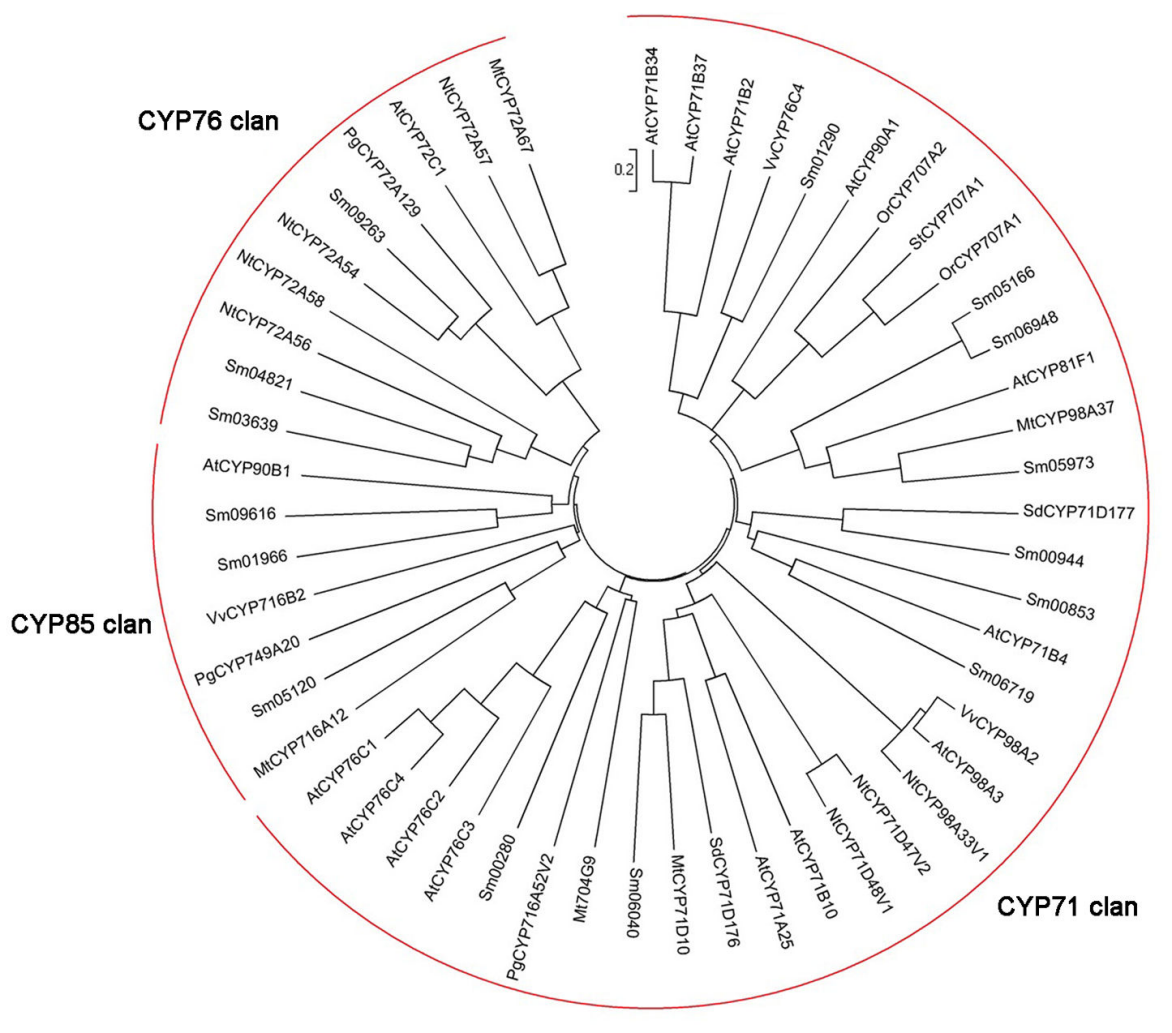
Phylogenetic analysis of CYP450s from *S. miltiorrhiza* and other plants. Amino acid sequences were aligned using CLUSTALW program, and evolutionary distances were calculated using MEGA4 software with the Poisson correction method. Nt, *Nicotiana tabacum*; Pg, *Panax ginseng*; Mt, *Medicago truncatula*; Vv, *Vitis vinifera*; Sd, *Scoparia dulcis*; Or, *Orobanche ramose*; St, *Solanum tuberosum*; Sm, *Salvia miltiorrhiza*. The GenBank/EMBL/DDBJ accession numbers of the sequences are ABC69417.1 (NtCYP72A54), AEY75218.1 (PgCYP72A129), ABC69414.1 (NtCYP72A57), ABC69422.1 (NtCYP72A58), ABC69419.1 (NtCYP72A56), ABC59075.1 (MtCYP72A67), ABC59086.1 (MtCYP98A37), XP_002283338 (VvCYP98A2), ABC69384.1 (NtCYP98A33V1), NP_189259.1 (AtCYP71B4), NP_195459.1 (AtCYP81F1), NP_182079.1 (AtCYP76C4), NP_182081.1 (AtCYP76C2), ADA70805.1 (SdCYP71D176), XP_003617706.1 (MtCYP71D10), NP_200536.3 (AtCYP71B10), NP_680107.1 (AtCYP71A25), XP_002266024.1 (VvCYP716B2), NP_850337.1 (AtCYP98A3), NP_850439.1 (AtCYP76C1), NP_172767.1 (AtCYP71B2), XP_002276576.1 (VvCYP76C4), NP_173149.1 (AtCYP72C1), XP_003592376.1 (Mt704G9), AEY75214.1 (PgCYP749A20), NP_196188.1 (AtCYP90A1), AFO63032.1 (PgCYP716A52V2), ABC59076.1 (MtCYP716A12), NP_190635.1 (AtCYP90B1), NP_182082.2 (AtCYP76C3), AFP74115.1 (OrCYP707A2), ABA55732.1 (StCYP707A1), AFP74114.1 (OrCYP707A1), ADA70806.1 (SdCYP71D177), ABC69395.1 (NtCYP71D47V2), ABC69397.1 (NtCYP71D48V1), NP_189261.1 (AtCYP71B34), NP_189264.3 (AtCYP71B37).

We then used 5’ and 3’ RACE (rapid amplification of cDNA end) to isolate full length cDNAs (with likely complete open reading frames) of 15 *P450* genes. Phylogenetic analysis of the deduced protein sequences with P450s from other plants revealed that 9 of them (Sm01290, Sm05166, Sm06948, Sm05973, Sm00944, Sm00853, Sm06719, Sm06040 and Sm00280) belong to the CYP71 clan, 3 (Sm05120, Sm01966 and Sm09616) to the CYP85 clan, and the rest 3 (Sm03639, Sm04821 and Sm09263) to the CYP72 clan (Figure 5). 

The CYP71 clan P450s are in the A-type P450 clade, and contains the most of P450 families involved in plant secondary metabolism, such as CYP73, CYP98, CYP76 and CYP706 families [35]. For example, the rice *CYP701A8* gene encodes an ent-kaurene oxidase that catalyses C3α-hydroxylation of *ent*-sandaracopimaradiene and *ent*-cassadiene, both are biosynthetic intermediates of the oryzalexin and the phytocassane families of phytoalexins [36]. In addition, a diterpenoid biosynthetic gene cluster was identified on rice chromosome 2, in which two genes encoding CYP71 clan members of CYP76M7 and CYP71Z7 catalyse the early and later steps in phytocassane biosynthesis, respectively [37,38]. Thus, the S. *miltiorrhiza* CYP71 clan members can be of particular interests in further elucidation of biosynthetic pathway of tanshinones. Due to distinct catalytic activities of P450 monooxygenases, differentially expressed *P450* genes of the CYP85 and CYP72 clans also hold the potential to be enzymes of the pathway. 

Biosynthesis of tanshinones involves downstream steps of decarboxylation, oxidation and reductions, which can be catalysed by decarboxylase, dehydrogenase and reductase, respectively [7,9]. The dehydrogenase-catalysed dehydration steps are likely involved in the conversion of ferruginol to cryptotanshinone, whereas the conversion of cryptotanshinone to tanshinone IIA requires a double band reduction at the position between C15-C16, which may be catalysed by a reductase (Figure 1). Unigene dataset was searched with *Zingiber zerumbet* short-chain dehydrogenase (ZSD1, AB480831) [39] and *Artemisia annua* aldehyde Δ11(13) reductase 2 (DBR2, EU704257) [40] to identify candidate dehydrogenases and reductases. One gene showed 66% nucleotide sequence identity to *ZSD1* (named *SmSD*) and another showed 69% identity to DBR2 (named *SmDBR*). Both genes were expressed at significantly higher levels in root than in leaf, with 15.5-fold of RPKM value (FDR < 0.01) for *SmSD* and 2.3-fold (FDR < 0.01) for *SmDBR* (Table 4), suggesting a high possibility of SmSD and SmDBR being involved in the biosynthesis of tanshinones.

### Prediction of transcription factors in tanshinones biosynthetic pathway

In plants, transcription factors of different families have been found to regulate secondary metabolism pathways, including those of WRKY, AP2/ERF, MYB and bHLH families [41,42]. In our unigene dataset, 735 genes were annotated as transcription factors, including zinc finger (148), AP2/ERF (82), MYB (82) and WRKY (61) family members. Among these, 209 were expressed in both root and leaf tissues, 67 showed a higher expression level in root and 45 in leaf (Table S4 and S5). Of the root up-regulated transcription factors, members of the AP2/ERF and GRAS families were markedly enriched, with 8 AP2/ERF genes up-regulated in root versus 2 in leaf, and 5 GRAS genes enriched in root versus only 1 in leaf (Table 5). Considering the root-specific accumulation of tanshinones, such higher expressions of these transcription factors in root suggest that they are worthy of further investigation. 

**Table 5:** Summary of transcription factor unigenes of *S. miltiorrhiza*.

**TFs family**	**Number of genes detected**	**Up-regulated in root**	**Up-regulated in leaf**
WRKY	61	8	6
MYB	82	8	6
AP2/ERF	82	8	2
MYC	9	0	2
GRAS	30	5	1
zinc finger	148	11	7
bHLH	42	5	4
bZIP	34	3	2
Others	247	19	15
Total	735	67	45

The AP2/ERF members have been shown regulate secondary metabolism pathways in several medicinal plants. In *A. annua*, two JA-responsive AP2/ERF proteins AaERF1 and AaERF2 positively regulate genes encoding amorpha-4,11-diene synthase (ADS, a sesquiterpene synthase) and CYP71AV1, two key enzymes of artemisinin biosynthesis [43]. In *Catharanthus roseus*, the AP2/ERF proteins of ORCA2 and ORCA3 regulate terpenoid indole alkaloid metabolism through binding to the promoter of the *strictosidine synthase* (*STR*) gene and activating its expression [44,45]. In present analysis, 82 unigenes were annotated as AP2/ERF family transcription factors, of which 8 were drastically abundant in root and thus may be related to root-specific production of tanshinones. 

Root plays a vital role in plant growth and development. The transcription factors of GRAS family, such as SHORT-ROOT (SHR) and SCARECROW (SCR), have been reported to play a critical role in root cortex development [46-48]. SHR protein acts as both a signal from the stele and an activator of endodermal cell fate whereas SCR mediats cortex cell division [49]. Moreover, it has been proposed that SHR and SCR have a conserved function in determining media cell fates and multiplication of cell layers in rice and Arabidopsis [50]. From the S. *miltiorrhiza* transcriptome, two genes were identified as SHR and SCR homologs, respectively. Tanshinones accumulate mainly in cortex of *S. miltiorrhiza* root, thus the identification of GRAS family factor genes expressed in root may provide an insight into the relation between cortex development and biosynthesis of tanshinones.

## Conclusions

This study generates the largest dataset of unigenes for *S. miltiorrhiza* and provides candidate enzymes involved in tanshinones biosynthesis based on their root-preferential expression pattern, including CYP450s, dehydrogenases and reductases. Candidate transcription factors were also identified, which are of great interests in further investigation of the relationship between biosynthesis of tanshinones and root development. Our data are also of great value to understanding the biosynthesis and regulation of secondary metabolites in perennial plants.

## Materials and Methods

### Plant tissue collection

Seeds of *Salvia miltiorrhiza* Bunge were collected from Anhui Province, China, and grown in field in Shanghai Chenshan Botanical Garden with approval of Shanghai Chenshan Botanical Garden. No protected plant species was sampled for this research. Leaf and root materials from 1-year-old plants were collected, immediately frozen in liquid nitrogen and stored at -80 °C prior to RNA extraction. 

### RNA extraction

Total RNAs from roots and leaves of three replicates were extracted using the TRIzol Reagent (Invitrogen) and treated with DNase I (Takara) according to manufacturer’s instructions. RNA quality was examined using 1% agarose gel and the concentration was determined using a Nanodrap spectrophotometer (Thermo). Root and leaf RNA pools were prepared by mixing equal amounts of three RNA replicates.

### cDNA library construction and 454 sequencing

Reverse transcription was performed with the SMART II^TM^ cDNA Synthesis Kit (Clontech, USA) according to manufacturer’s instructions. Double stranded cDNAs were separated on 2% agarose gel, and those >100 bp were recovered. Concentrations of cDNAs were determined by Bioanalyzer 2100 (Agilent, Germany), and subjected to pyrosequencing with the GS-FLX Titanium Instrument (Roche). Image and signal processing were performed using 454 Life Science software (Roche). All sequence data have been deposited to the National Center for Biotechnology Information’s Sequence Read Archive (SRA) with the accession number of SRR1005880.

### Assembly

To obtain high quality clean sequences, SeqClean was used to trim adapter sequences and Lucy (version 1.20p) was used to remove low quality sequences and those < 50 bp. Clean reads from root and leaf libraries were assembled into 16,806 isotigs and 84,116 singletons using Newbler (Roche, version 2.6) software. To reduce the redundancy, clustering was performed with CD-HIT (version 4.0), and isotig and singleton sequences with minimum 95% identity were merged into a single representative unigene. 

### Annotation of unigenes

Unigenes were used as query sequences to search against the non-redundant protein (NR) database at NCBI (http://www.ncbi.nlm.nih.gov) and the Swiss-Prot protein database (http://www.ebi.ac.uk/uniprot) with E-value cutoff of 1e^-5^. The annotations of the best hits were recorded. Gene Ontology (GO) (http://www.geneontology.org/) were further used to category the function of the unigenes by Blast2GO [51], and the unigenes were assigned to biological functions on the macro levels of “biological process”, “cellular component” and “molecular function”. The Kyoto Encyclopedia of Genes and Genome (KEGG) pathways database (http://www.genome.jp/kegg/) were assigned to unigenes by KEGG Automatic Annotation Server (KAAS) [52]. 

### Differential expression analysis

Gene expression levels of unigenes in roots or leaves were normalized and calculated as reads per kb per million reads (RPKM) values during the assembly and clustering process. Significance of differential gene expression between the root and leaf tissues was determined by a *p*-value < 0.05 by random test and corrected using the false discovery rate (FDR). 

### Determination of tanshinones by HPLC

Determination of tanshinones contents was carried out with an Agilent 1260 Infinity HPLC system as described [53], and equipped with a diode array detector (DAD, G4212B) using a SB-C18 Analytical HPLC Column (4.6 x 250 mm, 5 um, Agilent). The mobile phase consisted of 0.1% (vol/vol) formic acid methanol solution (A) and 0.1% (vol/vol) formic acid aqueous solution (B), with a gradient elution from 37% to 80% at a flow rate of 1.0 mL∙min^-1^. The HPLC chromatogram was monitored at 270 nm and the column temperature was set at 30 °C. The dihydrotanshinone I, tanshinone I, cryptotanshinone and tanshinone IIA were determined by comparing to authorized standards (WuXi App. Tec., China).

### Quantitative real-time RT-PCR

Total RNA were reverse transcribed with the ReverTra Ace -α- kit (Toyobo) according to manufacturer’s protocol. Real-time PCR was performed using the SYBR® Premix Ex Taq™ II (Perfect Real Time) kit (Takara) on a Mastercycler system (Eppendorf, Germany) with gene-specific primer pairs (Table S6). *ACTIN* was used as the internal reference gene [54]. The relative expression value was calculated via the 2^-ΔΔCt^ method [55].

### Full-length cDNA verification and cloning of CYP450s

The 5’ and 3’ RACE (rapid amplification of cDNA end) were performed by the 5’-Full RACE Kit and the 3’-Full RACE Core Set Ver.2.0 (Takara) according to manufacturer’s instructions. PCR products were cloned into the pMD18-T Vector (Takara) for Sanger sequencing. 

Full-length cDNAs were obtained by assembling fragments obtained by 5’ and 3’ RACE in combination with annotated unigene sequences. P450 sequences from other plant species were obtained by searching the GenBank database at NCBI. The amino acid sequence alignment of P450 proteins was performed with CLUSTALW program of MEGA 4 software with default parameters. Phylogenetic tree was built with the neighbour-joining method with MEGA 4 program.

## Supporting Information

Figure S1 **HPLC analyses of tanshinones in *S. miltiorrhiza* root and leaf.** *S. miltiorrhiza* root (A) and leaf (B).
(TIF)

Table S1 **Functional classification of metabolic pathways related to secondary metabolism by KEGG.**
(XLSX)

Table S2 **List of unigenes expressed more abundantly in *S. miltiorrhiza* leaf.**
(XLSX)

Table S3 **List of unigenes that are more abundant in *S. miltiorrhiza* root.**
(XLSX)

Table S4 **Transcription factors that showed higher expression levels in *S. miltiorrhiza* root.**
(XLSX)

Table S5 **Transcription factors that showed higher expression in *S. miltiorrhiza* leaf.**
(XLSX)

Table S6 **List of oligonucleotide primer sequences.** All primers used for full-length cDNA cloning and qRT-PCR analyses of P450s in present study are listed.
(XLSX)
