# Correction: Transcriptome Analysis of Medicinal Plant *Salvia miltiorrhiza* and Identification of Genes Related to Tanshinone Biosynthesis

**DOI:** 10.1371/annotation/fd65b655-d35b-47d1-8793-07da2273c144

**Published:** 2013-12-16

**Authors:** Lei Yang, Guohui Ding, Haiyan Lin, Haining Cheng, Yu Kong, Yukun Wei, Xin Fang, Renyi Liu, Lingjian Wang, Xiaoya Chen, Changqing Yang

The name of the ninth author is spelled incorrectly. The correct name is: Lingjian Wang 

