# Supplementary figures and images for: Transcriptome Analysis of Medicinal Plant Salvia miltiorrhiza and Identification of Genes Related to Tanshinone Biosynthesis
Source: PLoS One. 2013 Nov 19;8(11):e80464. doi: 10.1371/journal.pone.0080464 (PMC3834075; doi:10.1371/journal.pone.0080464)

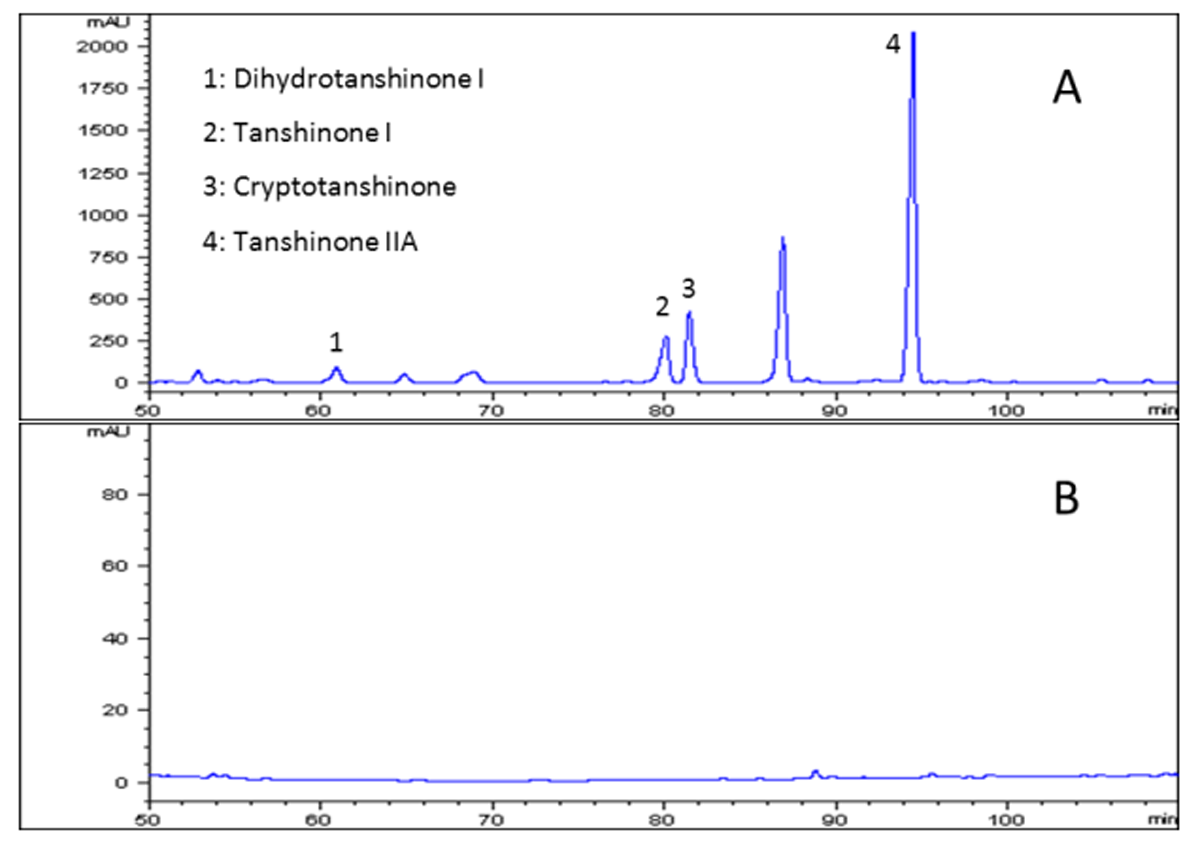

Supplement: Figure S1 — HPLC analyses of tanshinones in S. miltiorrhiza root and leaf. S. miltiorrhiza root (A) and leaf (B). (TIF) [file pone.0080464.s001.tif]
